# Analysis of clinical malaria hotspots according to transmission levels in two health areas of the Kolondieba health district, Mali

**DOI:** 10.5281/zenodo.19822964

**Published:** 2026-05-02

**Authors:** Ibrahima Berthé, Mady Cissoko, Modibo Diarra, Donatien Serge Mbaga, Mamady Koné, Ahmadou Boly, Ousmane Boua Togola, Bayaya Haidara, Ismaila Théra, Abdoulaye Ongoiba, Tahirou Togola, Amagoron dit Mathias Dolo, El Hadji Issa Amaguiré Sy, Abdramane Konaté, Youssouf Coulibaly, Lassana Sissoko, Issa Bougoudogo, Bakary Niangado, Abdou Togola, Dramane Traore, Leon Paul Rabarijaona, Kalifa Keita, Bouyagui Traoré, Cheick Amadou Tidiane Traoré, Issaka Sagara

**Affiliations:** 1 Disease Prevention and Control Department and General Directorate of Health and Public hygiene, Ministry of Health and Social Affairs, Bamako, Mali.; 2 Parasites and Microbes Research and Training Center, Department of Epidemiology of Parasitic Diseases, Bamako, Mali.; 3 National Malaria Control Program, Bamako, Mali; 4 Department of Biomedical Sciences, Faculty of Sciences, The University of Bertoua, Bertoua, Cameroon.; 5 Institut National de Santé Publique, Mali.; 6 Kolondieba District Hospital, Kolondieba, Mali.; 7 Kadiana Community Health facility, Kolondieba, Mali.; 8 Kolondieba central Community Health facility, Kolondieba, Mali.; 9 Fakola Community Health facility, Kolondieba, Mali.; 10 Sikasso Regional Health Directorate.; 11 Unicef Mopti Field Office, Mali.

## Abstract

**Background:**

Despite significant progress, malaria remains a major challenge in Mali, due to heterogeneous transmission. The WHO ‘High Burden to High Impact’ strategy advocates targeting of high-transmission areas. This study aimed to identify clinical malaria hotspots in two health areas within the Kolondieba district with differing transmission levels.

**Materials and Methods:**

A retrospective cross-sectional study analysed 35,934 confirmed malaria cases extracted from consultation registers between 2019 and 2021 in the health areas of Kadiana (high risk) and Kolondieba Central (moderate risk). Transmission periods were determined in each zone by analysing variations in mean incidence trends, and high-risk clusters (hotspots) were identified using Kulldorff’s method according to these periods.

**Results:**

Transmission periods and hotspot identification (2019–2021) revealed distinct dynamics between the two health areas. In Kadiana, the majority of clusters remained stable, with persistent hotspots across all eight periods (Kadiana and Tienkourani; RR: 1.8–6.3). In Kolondieba Central, hotspot dynamics evolved from a localised configuration (1 to 2 villages) towards spatial expansion, with more extensive clusters appearing during the final periods (RR: 1.7–2.2). Kolondieba town remained a persistent hotspot.

**Conclusions:**

This study confirms the heterogeneity of transmission at a fine scale, with stable hotspots even in both moderate and high-risk areas. It is essential to focus on human mobility to guide malaria control interventions.

## Introduction

Malaria represents a major global public health problem, with strong persistence in sub-Saharan Africa. According to the latest world malaria report report, there were an estimated 282 million cases and 610,000 fatalities globally with 95% of this burden concentrated in the WHO African Region [1].

Mali remains one of the eleven countries most severely impacted by malaria, accounting for a significant proportion of the global burden. Within the domestic clinical landscape, malaria represents the primary aetiology for outpatient consultations across various healthcare facilities [2,3]. Despite national efforts, including the malaria strategic plan (2018–2022) and adherence to the WHO ‘High Burden to High Impact’ approach, the country has recorded an increase in disease incidence in recent years, exacerbated by vector resistance, security instability, and funding deficits [4,5].

Despite significant progress, achieving the goals of the Global Technical Strategy for Malaria (2016–2030) which aims for a 90% reduction in incidence and mortality is complicated by factors such as insecticide resistance, limited access to health services, socio-political conflict, and the effects of climate change [6,7]. The complex spread of malaria is defined by significant geographical and seasonal variability. This phenomenon is primarily driven by specific meteorological parameters, including ambient temperature, precipitation and atmospheric humidity, alongside ecological variables such as the availability of larval habitats and patterns of land utilisation. Additionally, anthropogenic influences, ranging from socio-economic disparities and access to healthcare to urbanisation and land tenure, play a critical role in shaping the epidemiological landscape of the disease [7–9]. This complexity makes it imperative to identify areas where malaria transmission persists. These areas, often characterised by a concentrated parasite burden, are fundamental to optimising the deployment of interventions and maximising the effectiveness of control strategies, particularly within the framework of the tailored and targeted approaches advocated by the Global Technical Strategy for Malaria 2016–2030 [10,11].

The district of Kolondieba, located in Sikasso region of southern Mali, represents a particularly affected endemic zone, with high incidence and pronounced vulnerability among children 0-4 years of age [12]. A preliminary spatial analysis revealed risk disparities within this district, identifying health areas with moderate and high transmission [12]. However, the hypothesis that risk heterogeneity persists at a finer scale (village) suggesting the existence of transmission foci (hotspots) within health areas classified as moderate or high transmission requires verification. This study aimed to identify and analyse clinical malaria hotspots in two health areas of the Kolondieba health district, exhibiting moderate and high endemicity from 2019 to 2021. By evaluating fluctuations in seasonal transmission windows, high-risk settlements, and vulnerable demographic cohorts, the research aims to refine vector control interventions and streamline the distribution of constrained resources.

## Materials and Methods

**Figure 1:**
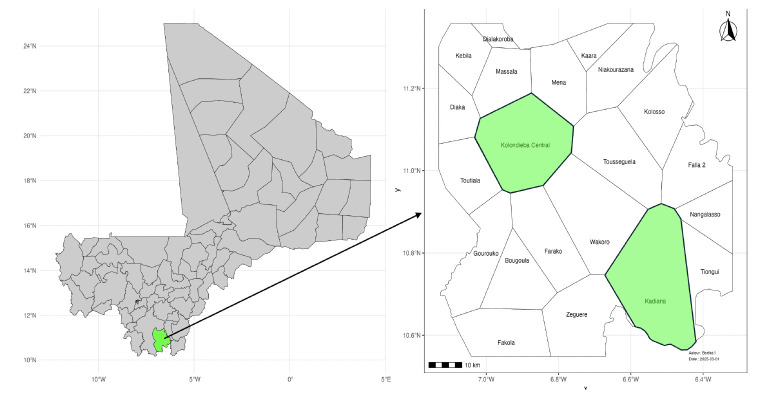
Kolondieba district in Mali (left, green) and the district’s health areas (right; Kolondieba Central and Kadiana highlighted in green).

This retrospective cross-sectional study, spanning from 2019 to 2021, was conducted in two health areas with differing malaria transmission risks [12], located in the Kolondieba health district, a malaria-endemic zone in southern Mali (Figure 1).

The first health area, Kadiana, is situated 60 km south of Kolondieba Central. This area, classified as high-risk for malaria transmission, comprises 22 villages. It borders Côte d'Ivoire, is crossed by a river, and hosts gold-mining sites. Kadiana had an estimated population of 34,457 in 2021. The second health area, Kolondieba Central, corresponds to the district capital and comprises 27 villages. It is located in an area classified as moderate-risk for malaria transmission, with an estimated population of 47,220 in 2021[12].

### Study population and inclusion criteria

The entire resident population of the Kadiana and Kolondieba Central health areas during the 2019–2021 period constituted the study population. All cases of clinical malaria confirmed by rapid diagnostic test (RDT) or thick smear microscopy, with complete records documented in the consultation registers of the Kadiana and Kolondieba Central health areas were included.

### Data collection

Data were extracted at the village level and included:

*Malaria cases*: Data from health centre registers (2019–2021). Only fully documented cases (age, sex, origin, clinical signs, RDT results, treatment administered) were included.

*Demographic data*: Extracted from the district health map, based on the 2009 General Population and Housing Census (RGPH), updated according to the district's annual growth rate [14].

*Meteorological parameters*: Remote sensing data obtained via Giovanni platform from National Aeronautics and Space Administration (NASA). Measurements were spatialised by health area using Voronoi polygons for precise attribution. The variables collected and their characteristics are shown in Table 1.

*Environmental characteristics*: Including hydrography, gold-mining areas, and accessibility to healthcare. These data were collected via a field survey among local stakeholders with triangulation of multiple sources.

**Table 1:** Meteorological variables and sources.

Variables	Timescale	Units	Sources	Spatial Resolution
Late cumulative precipitation	Daily	mm/day	GPM	0.1°
Mean soil moisture	Daily	Kg/m2	GLDAS Model	0.25°
Maximum temperature	Daily	°C	MERRA-2	0.5 x 0.625°
Mean temperature	Daily	°C	MERRA-2	0.5 x 0.625°
Minimum temperature	Daily	°c	MERRA-2	0.5 x 0.625°

### Data quality

To ensure accuracy of the epidemiological data, emphasis was placed on primary source data, which were subject to quality control through regular supervision, analysis sessions, and six-monthly validation by the health system. The KoboCollect application from manufacturer KoBoToolbox [15], was used by two enumerators experienced in using primary sources to collect data via a structured questionnaire to minimise human error. During processing, any inconsistencies or missing data were verified by cross-referencing with monthly reports from health facilities. The source of the meteorological information obtained is recognised for the reliability of its satellite archives.

### Data analysis

For each health area (Kadiana and Kolondieba Central), we constructed weekly time series including: (1) malaria incidence (number of confirmed cases relative to the population at risk, expressed as cases per 1,000 inhabitants), (2) mean temperature (in °C), (3) mean of soil moisture (Kg/m^2^), and (4) cumulative precipitation (mm). These indicators, aggregated at the level of each health area, were systematically aligned on a common timeframe, checked for completeness, and then integrated into a database for analysis.

To determine malaria transmission periods, a changepoint analysis (cpt) was applied to the weekly time series of malaria incidence (cases/ 1,000 inhabitants) for the Kadiana and Kolondieba Central health areas (2019–2021). This approach was performed using the Pruned Exact Linear Time (PELT) algorithm to detect statistically significant changes in the mean incidence. This allowed us to identify transmission periods that characterise the seasonal dynamics of malaria [16].

Weekly meteorological parameters (mean temperature in °C, cumulative precipitation in mm, mean of soil moisture in Kg/m^2^) were compared between the Kadiana and Kolondieba Central health areas over the study period. Comparisons were based on weekly mean values over the three years. The difference in means between the two zones was calculated for each parameter to assess discrepancies.

The choice of statistical tests was determined by the data distribution and compliance with application conditions. For comparisons of means: Student’s t-test (if normality conditions were met) or the non-parametric Kruskal-Wallis test as an alternative. For comparisons of proportions: Pearson’s χ^2^test or Fisher’s exact test for small sample sizes. The significance threshold was set at p < 0.05.

The mean annual incidence per village was calculated by dividing the sum of the annual incidences (expressed per 1,000 inhabitants) by the number of study years (n=3). The results were represented graphically using histograms, stratified by health area.

The villages presenting case aggregation were identified as high-risk clusters. These clusters were subsequently classified as hotspots. For each transmission period determined by the significant change in the mean weekly incidence for the years 2019, 2020, and 2021, a spatial analysis using Kulldorff’s method was performed to identify high-risk clusters. Population data, the number of malaria cases, and the geographic coordinates of each village were used in the analysis.

This analysis used circular windows centred on villages, with a radius varying from 1% to 50% of the total population. The algorithm detected areas with a significant excess of cases inside these windows compared to outside. The relative risk associated with each cluster was calculated as the ratio of the incidence rate inside the window to that outside, thereby quantifying the intensity of the spatial risk. Due to the counting data, a Poisson model was used, and Monte Carlo simulations were employed to assess the significance of each cluster.

Mean weekly incidences (per 1,000 inhabitants) were calculated by age group (0–4, 5–9, 10–14, 15–24, and ≥ 24 years) at the health area level over the 2019–2021 period. For each zone (high and moderate risk), a comparative analysis was performed to compare incidences between the different age groups. Comparisons were carried out using Student’s t-test (after checking normality conditions) or, failing that, the Kruskal-Wallis test, with a significance threshold of p < 0.05.

Analyses were performed using R (version 3.6.1) for statistical processing, SaTScan for cluster detection, and mapping of results. QGIS software (version 3.10) was used to produce the Voronoi polygons of the villages using a random method.

### Ethical considerations

The protocol for this study was approved by the Ethics Committee of the Faculties of Medicine and Odontostomatology, and of Pharmacy at the University of Sciences, Techniques and Technologies of Bamako (USTTB) under No. 2020_252_CE/ FMOS/FAPH. The health and administrative authorities of Kolondieba were informed of the protocol's content, and the Chief Medical Officer of the Kolondieba health district gave written consent for the collection and use of these data.

## Results

This study included 35,934 confirmed cases of malaria, with 10,653 and 25,281 cases from the Kolondieba Central and Kadiana health areas, respectively. These were extracted from consultation registers for the years 2019, 2020, and 2021.

**Figure 2:**
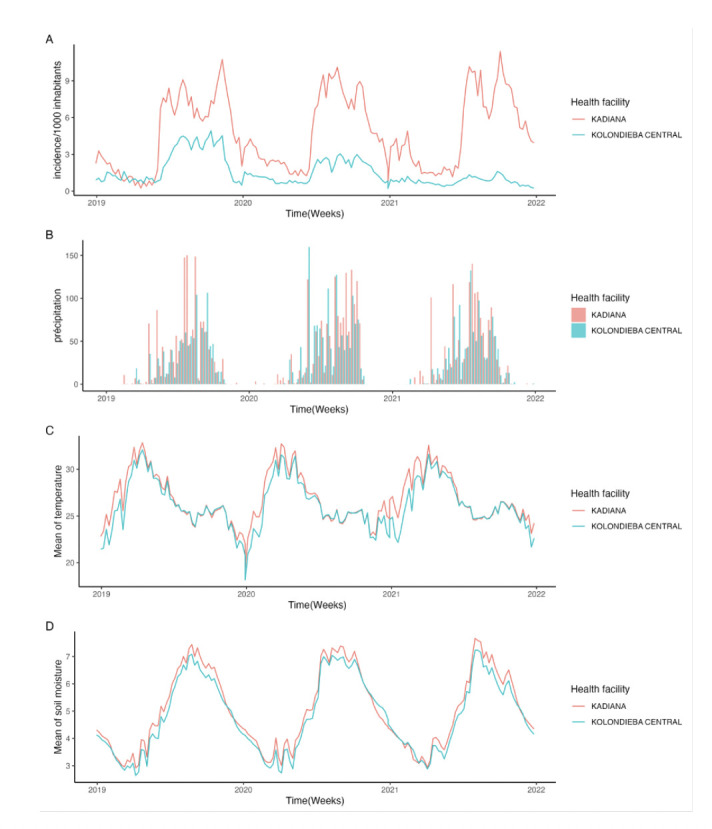
Weekly time series of malaria incidence and meteorological variables (rainfall, soil moisture, temperature) in the health areas of Kadiana (high risk) and Kolondieba Central (moderate risk), 2019–2021.

Figure 2 illustrates the temporal dynamics of malaria incidence and meteorological variables (rainfall, mean temperature, mean of soil moisture) on a weekly scale throughout the study period. Over the 2019–2021 period, the mean weekly incidence was high in Kadiana at 4.7 per 1,000 inhabitants, compared to 1.5 per 1,000 inhabitants in central Kolondieba. The progression of incidence follows a trend similar to that of rainfall and soil moisture, but appears to move in the opposite direction to variations in mean temperature.

In the Kadiana health area, meteorological factors appear slightly higher than those in the Kolondieba Central health area, which is situated in a moderate-risk zone. Average weekly rainfall was higher, with mean annual totals (2019–2021) of 1,474 mm in Kadiana versus 1,219 mm in Kolondieba Central. The maximum weekly temperature reached a peak of 41.6 °C in Kadiana, exceeding the maximum of 40.4 °C recorded in Kolondieba Central. Finally, the mean weekly soil moisture was slightly higher in Kadiana, with a difference ranging from 1.0-1.5 percentage points between the two sites.

**Figure 3:**
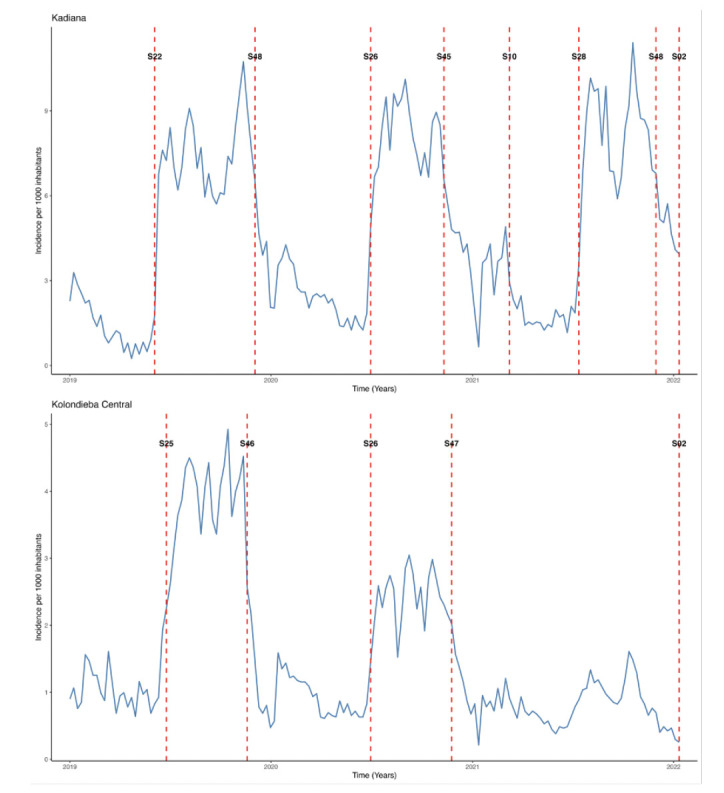
Determination of malaria transmission periods using change-point analysis of mean incidence from 2019 to 2021 in Kadiana (high risk) and Kolondieba Central (moderate risk). ’S’ indicates the week number.

Between 2019 and 2020, the Kadiana health area exhibited two periods of high transmission, characterised by a weekly incidence ranging from 6-10 cases per 1,000 inhabitants (Figure 3). These periods spanned from week 22 to week 47 in 2019, and from week 26 to week 44 in 2020. In contrast, Kolondieba Central, a moderate-risk zone, showed slightly shifted high-transmission periods (ranging from 2-5 cases per 1,000 inhabitants per week): from week 25 to week 45 in 2019, and from week 26 to week 46 in 2020. In 2021, the divergence between the two zones became more pronounced.

Kadiana presented three distinct transmission periods: a low phase from week 10 to 27, a high phase from week 28 to 47, and a moderate phase from week 48 to 52. Conversely, Kolondieba Central showed only a single transmission period, which was generally low with no marked peak.

The weekly average of the meteorological parameters was comparable between the two health areas over the period, with the exception of temperature, where a statistically significant difference (p = 0.04) was noted (Table 2).

**Table 2:** Comparison of the weekly mean of specific meteorological parameters in the health areas of Kadiana and Kolondieba Central from 2019 to 2021.

Variable	Health Area		Mean Difference	P-value
	Kadiana	Kolondieba Central		
Mean weekly precipitation	27.81	22.98	4.81	0.17
Mean weekly temperature	26.79	26.07	0.72	0.04
Mean weekly soil moisture	5.03	4.82	0.21	0.14

**Figure 4:**
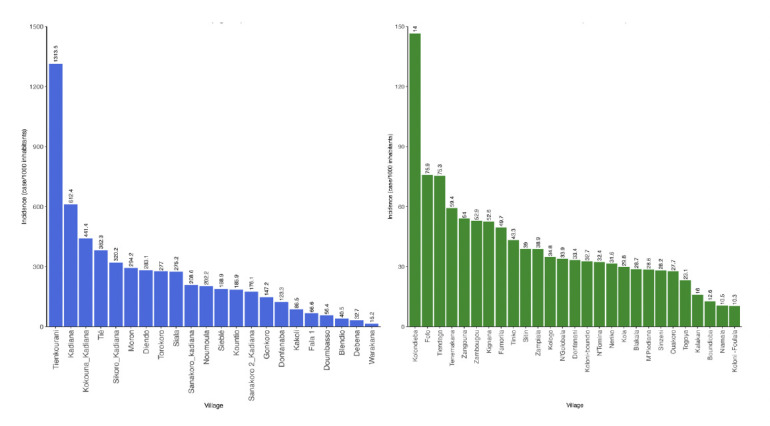
Mean incidence (2019–2021) of clinical malaria stratified by village in Kadiana (left) and Kolondieba Central (right). Note difference in y-axis scale.

A geographical disparity at the village level was observed in both high-risk and moderate-risk areas. In the high-risk health area, the mean incidence (2019–2021) ranged from 15 to 1,313 cases per 1,000 inhabitants, compared to 5 to 147 cases per 1,000 inhabitants in the moderate-risk area (Figure 4).

The villages in the Kadiana health area with the highest incidences were Tiekourani, Kadiana, and Tie, with 1,313 cases, 441 cases, and 382 cases per 1,000 inhabitants respectively. Conversely, in the Kolondieba Central health area, incidences were lower, with a notable exception in the village of Kolondieba home to the referral health centre which shows a higher incidence (147 cases per 1,000 inhabitants) than its neighbours.

## Determination of malaria high-risk clusters (hotspots)

**Figure 5:**
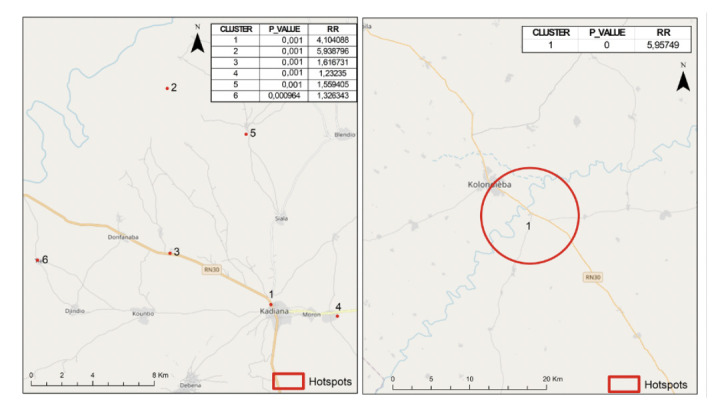
Malaria clusters in the Kadiana health facility (left) with six clusters of one village each (Kadiana = 1, Tienkourani = 2, Sikoro = 3, Moron = 4, Kokouna = 5, Tié = 6) and the Kolondieba Central health facility (right) with one cluster of two villages (Kolondieba town and Tiendaga).

The maps in Figure 5 display malaria transmission hotspots within the two health areas characterised by moderate and high transmission in 2021. On the left (Kadiana health area - high transmission), six significant clusters were identified, each corresponding to a specific village. Relative Risk (RR) values range from 1.23 to 5.93, all with very low p-values (p < 0.001), indicating high statistical significance. These include the villages of Kadiana (RR = 4.10, p < 0.001), Tienkourani (RR = 5.94, p < 0.001), Sikoro (RR = 1.62, p < 0.001), Moron (RR = 1.23, p < 0.001), Kokouna (RR = 1.56, p < 0.001), and Tie (RR = 1.33, p < 0.001).

On the right (Kolondieba Central health area - moderate transmission),Oonly one significant cluster was detected, encompassing two adjacent villages (Kolondieba Town and Tiendaga). This cluster presents a high relative risk (RR = 5.95) with a low p-value (p < 0.001).

**Figure 6:**
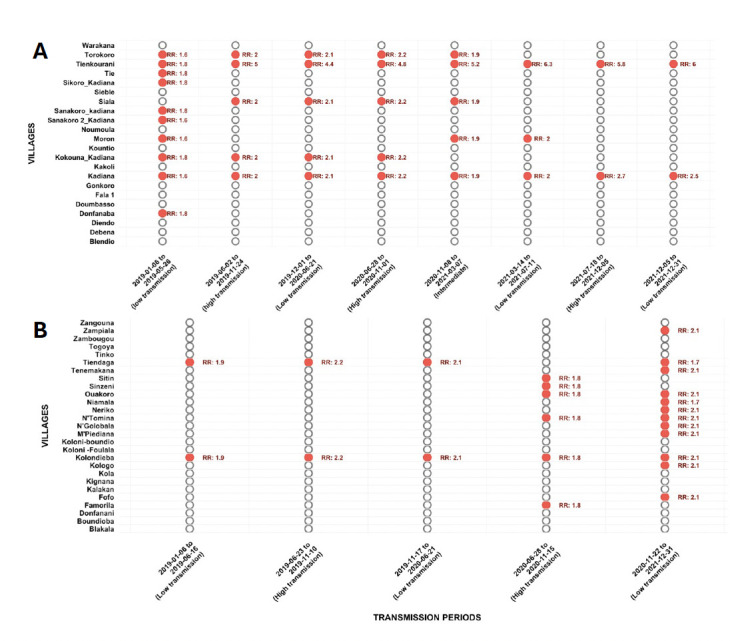
Spatio-temporal evolution of high risk clusters identified in the Kadiana (A, high risk) or Kolondieba Central (B, moderate risk) health areas. Villages with the same relative risk over a given period constitute the spatial entity of a cluster. Red dots depict significant clusters (RR = Relative Risk values).

Throughout the entire study period (2019–2021), at least one transmission cluster comprising one or more villages was identified during each of the defined transmission periods, with relative risks ranging from 1.8 to 6.3. Out of the 22 villages in the study area, 11 (50%) were part of a cluster during at least one period. Certain villages demonstrated remarkable temporal persistence: for instance, Tienkourani and Kadiana remained active hotspots throughout the eight successive periods (Figure 6A). In the Kolondieba Central health area, only small clusters were observed during the first three periods, with just a single grouping of two villages per period. This dynamic subsequently changed during the final two periods, which saw the emergence of more extensive clusters, encompassing 6 to 12 villages out of the 27 in the health area. Despite this evolution, only the village of Kolondieba remained a persistent hotspot, being included in a cluster across all five periods with a moderate relative risk (RR: 1.7-2.2) (Figure 6B).

For Kadiana, the 0–4 yrs age group experienced a significantly higher incidence than all other age groups (Table 3). The 5–9 yrs group had a higher incidence than the older groups, but lower than the 0–4 yrs group. Only the 10-14 yrs and 25+ yrs groups showed no significant difference (Table 3). For Kolondieba Central, the 0–4 yrs age group had a significantly higher incidence than all other groups (p < 0.05). The 5–9 yrs group had a higher incidence than the older groups, but lower than the 0–4 yrs group. Only the 10-14 yrs and 15-24 yrs groups showed no significant difference (Table 3).

**Table 3:** Comparison of average weekly incidence (2019–2021) by age group in the Kadiana health area (high risk) and Kolondieba Central (moderate risk).

	Kadiana				Kolondieba Central			
Age group (yrs) comparison	Group 1	Group 2	Mean Difference	P-value	Group 1	Group 2	Mean Difference	P-value
0-4 vs 5-9	11.2	5.9	5.3	<0.0001	3.2	1.9	1.3	<0.0001
0-4 vs 10-14	11.2	2.4	8.8	<0.0001	3.2	1.3	1.9	<0.0001
0-4 vs 15-24	11.2	3.0	8.2	<0.0001	3.2	1.1	2.1	<0.0001
0-4 vs 25+	11.2	2.5	8.7	<0.0001	3.2	0.7	2.5	<0.0001
5-9 vs 10-14	5.9	2.4	3.5	<0.0001	1.9	1.3	0.6	0.0007
5-9 vs 15-24	5.9	3.0	2.9	<0.0001	1.9	1.1	0.8	<0.0001
5-9 vs 25+	5.9	2.5	3.4	<0.0001	1.9	0.7	1.2	<0.0001
10-14 vs 15-24	2.4	3.0	0.6	0.0063	1.3	1.1	0.2	0.0859
10-14 vs 25+	2.4	2.5	0.1	0.4653	1.3	0.7	0.6	<0.0001
15-24 vs 25+	3.0	2.5	0.5	0.0282	1.1	0.7	0.4	<0.0001

### Discussion

Our research on the analysis of clinical malaria hotspots in the Kolondieba Health District, conducted from 2019 to 2021, revealed complex epidemiological dynamics characterised by spatial heterogeneity and significant variations across age groups.

A total of 35,934 confirmed malaria cases (excluding those from outside the catchment areas) were recorded over the 3-year period, representing an annual incidence 152 cases per 1,000 inhabitants. A notable concentration of cases was observed in the Kadiana health area (25,281 cases; 251 per 1,000) compared to Kolondieba Central (10,653 cases; 78 per 1,000), highlighting the persistence of a significant malaria burden within the district.

These general figures align with the conclusions of Zhang *et al.* [17], who, despite a global decline in the malaria burden between 1990 and 2021, insist that malaria remains a serious public health threat, particularly in sub-Saharan Africa. The high incidence in our study area (152 per 1,000), justifies the need for targeted interventions [17].

### Time-series analysis of incidence and climate data

The analysis reveals that climate parameters (rainfall, temperature, humidity) are slightly more favourable in the Kadiana area, classified as high-risk, than in Kolondieba Central, classified as moderate-risk. These minor environmental differences do not align with the mean weekly incidence observed over the same period, which was significantly higher in Kadiana (4.7%) than in Kolondieba Central (1.5%). This narrow gap between the meteorological profiles of the two zones raises questions about the relative weight of climatic factors in current risk stratification. Our observations are consistent with the literature [18] highlighting that while climatic parameters provide a broad framework, their ability to predict risk at an operational scale is limited by local landscape heterogeneity [19], intervention coverage, and socio-behavioural determinants.

### Determination of malaria transmission periods: High-risk vs moderate-risk zones (2019–2021)

Our results highlight a temporal heterogeneity in malaria transmission periods, closely linked to the risk level. In 2021, the Kadiana zone experienced three distinct transmission periods (low, moderate, and high), while only one low-transmission period was observed in the moderate-risk zone (Kolondieba Central). This disparity is consistent with the work of Lubinda *et al.* [20] in Zambia and Cissoko *et* al. [21] in Mali, who also reported significantly variable spatio-temporal trends between district and finer (health area) levels. This observation aligns with the distinction made by Stresman *et al.* between ‘stable hotspots’ and ‘unstable hotspots’ [22]. Kadiana's profile, with multiple transmission phases, corresponds more closely to the notion of an unstable hotspot, characterised by ephemeral peaks linked to seasonal transmission [22]. This temporal granularity, often masked in district-level analyses [12], highlights the need for high-resolution spatio-temporal surveillance to adapt interventions to local dynamics.

### Comparative analysis of meteorological parameters between health areas (2019–2021)

Our results suggest that mean weekly temperature is the primary meteorological factor discriminating between the two zones. This difference could help explain the more sustained level of transmission observed in Kadiana, in accordance with the work of Yamba *et al.* [23] and Yao *et al.* [24], which highlights the decisive influence of temperature on parasite and vector dynamics. However, the similarity in rainfall patterns and soil moisture between the two zones indicates that climatic factors alone do not fully explain the risk gradient. Thus, while temperature may serve as a useful predictive indicator for anticipating periods of high transmission, it must be integrated into multidisciplinary models including local socio-economic, behavioural, and environmental determinants. The complexity of these interactions is also justified by Atusingwize *et al.* [25] and Khazaee-Pool *et al.* [26], who emphasise that specific micro-environmental conditions (exposure, vegetation cover, housing type) can amplify or mitigate the effect of average climatic variables.

### Mean incidence by village (2019–2021) stratified by health area

Our analyses revealed a spatial heterogeneity in malaria incidence at the village level in both the high and moderate risk areas. These observations corroborate those reported by Zhang *et al.* [17], who highlight local variations in the malaria burden with higher incidences in certain parts of sub-Saharan Africa. Similarly, Yao *et al.* [24] highlight the existence of geographical disparities, spatial autocorrelation, and heterogeneity in malaria patterns across sub-Saharan Africa. Our results provide concrete evidence of this heterogeneity at a finer local scale (village), which is crucial for targeted interventions. Thus, our results justify the need for a geospatial approach to malaria surveillance and control, as advocated by Odhiambo *et al.* [27], who state that ‘high-resolution maps revealing the spatio-temporal variation of malaria endemicity are useful for estimating the malaria burden’. Atusingwize *et al.* [25] also corroborate this heterogeneity by insisting on the importance of local social and environmental determinants.

### Identification of malaria hotspots

Spatial analysis using Kulldorff's scan statistic identified six significant clusters in 2021, each corresponding to a village in the high-transmission Kadiana health area. The presence of several isolated clusters suggests sustained malaria transmission in this border zone. As Kadiana borders Côte d'Ivoire, it experiences significant population flows. These movements facilitate the introduction and spread of parasite strains, as confirmed by COVID-19 studies where clusters were associated with high-mobility areas [8].

The continuous presence of clusters in the Kadiana area across the eight transmission periods from 2019 to 2021 highlights active transmission throughout the study. The persistence of Tienkourani and Kadiana as clusters over all eight periods suggests the existence of stable endemic foci, requiring targeted and sustainable interventions. Conversely, the situation in Kolondieba Central in 2021, where only one significant cluster was identified (encompassing two neighbouring villages), indicates more localised and contained transmission. The detection of this single focus, despite presenting a high risk, suggests a close epidemiological connection between these two communities. This dynamic could be attributed to shared socioenvironmental conditions (such as similar agricultural practices). Cluster identification analysis by transmission period (5 periods) in Kolondieba Central found a single cluster grouping two localities (Kolondieba town and Tiendaga village) during the first three periods, followed by a 6-village cluster and then two 12-village clusters in the 4^th^ and 5^th^ periods, respectively. This progression suggests that the neighbouring localities (Kolondieba town and Tiendaga) constitute a stable endemic focus from which transmission periodically spreads to other villages. It should be noted that the relativity between villages within the same transmission zone can lead to the identification of clusters that reflect intra-zone disparities rather than fundamental epidemiological differences.

In Kolondieba town, the majority of case confirmations are performed via thick smear film, a technique whose sensitivity and specificity are operator-dependent. This could influence case detection and potentially overestimate the incidence in Kolondieba town, contrasting with the literature's expectations of lower incidence in urban environments.

To contain this dynamic, a two-level control strategy should be indicated: sustained interventions targeting the endemic core, combined with reinforced surveillance during spatial expansion phases.

### Comparison of mean weekly incidences (2019–2021) by age group

The analysis of mean weekly incidence confirms the predominant vulnerability of children aged 0–4 yrs in both health areas. Incidence then progressively decreases with age, a profile consistent with the development of acquired immunity following repeated exposure in endemic areas. These results align with epidemiological observations in sub-Saharan Africa, where the malaria burden has historically been concentrated in young children [12, 13]. However, the magnitude of this difference could be partly attributable to a detection bias linked to the free of charge policy. Free malaria care for children under five encourages early diagnosis and consultation, increasing the number of recorded cases in this group, whereas the incidence in older age groups (not eligible for free care) may be underestimated due to lower healthcare-seeking rates. These results contrast with profiles reported in some low-transmission regions or areas with different epidemiology where infection peaks may occur in older groups [26], thus highlighting the importance of the local epidemiological context in interpreting incidence data. By providing finegrained disaggregation by age and health area, this analysis complements macro-regional studies focused on climatic determinants or aggregated trends [23]. It offers an operational basis for targeting interventions notably the distribution of insecticide-treated nets, early diagnosis, and preventive treatment towards the most at-risk age group, while reinforcing the idea that control strategies must be adapted to the demographic and epidemiological specificities of each zone.

Although our results provide a detailed analysis, it is crucial to recognise gaps and pathways for improvement, particularly by integrating the dimension of human population movement (HPM). Tam *et al.* [28] highlight a lack of evidence regarding HPM tracking, which nonetheless remains a major obstacle to malaria control and elimination. Incorporating these data into future studies could provide a deeper understanding of the transmission dynamics underlying our hotspots, allowing for more effective targeted interventions.

### Limitations

Clinical malaria cases from private facilities were not collected due to insufficient or improper archiving of consultation registers. Meteorological data could not be obtained at a finer resolution.

## Conclusions

Our work has highlighted the spatio-temporal heterogeneity of malaria at the health area level in the Kolondieba health district, identifying stable and unstable hotspots at the village scale and significant variations by age group. These results, corroborated and justified by existing scientific literature, underscore the complexity of malaria transmission and the importance of targeted, granular approaches. Future research avenues, particularly the integration of human population movement data, promise to refine our understanding and improve malaria control strategies.

## References

[1] World Health Organization: World Malaria Report 2025. https://tinyurl.com/2du6s9ts.

[2] Ministère de la Santé et du développement social du Mali: Système national d’information sanitaire et social, 2022.. https://tinyurl.com/2ukm6dn4.

[3] World Health Organization: World Malaria Report 2023.. https://tinyurl.com/xd6w6z37.

[4] Cissoko M, Sagara I, Guindo A, Maiga M (2025). Impact of control interventions on malaria incidence in the general population of Mali.. J. Epidemiol. Glob. Health.

[5] Koenker H, Coulibaly MK, Bouare I Trends in and factors associated with malaria prevention in Mali: Further analysis of the Mali demographic and health surveys and malaria indicator surveys 2006-2018.. DHS Further Analysis Reports No. 132. Rockville, Maryland, USA: ICF..

[6] Leal Filho W,, May J,, May M,, Nagy GJ (2023,). Climate change and malaria: some recent trends of malaria incidence rates and average annual temperature in selected sub-Saharan African countries from 2000 to 2018.. Malar. J..

[7] Venkatesan P The 2023 WHO World malaria report.. Lancet Microbe 2024,.

[8] Martonik R, Oleson C, Marder E: (2024). Spatiotemporal cluster detection for COVID-19 outbreak surveillance: Descriptive analysis study.. JMIR Public Health Surveill..

[9] Li J, Docile HJ, Fisher D, Pronyuk K, Zhao L: (2024). Current status of malaria control and elimination in Africa: Epidemiology, diagnosis, treatment, progress and challenges.. J. Epidemiol. Glob. Health.

[10] Coulibaly D, Travassos MA, Tolo Y, Laurens MB (2017). Spatio-temporal dynamics of asymptomatic malaria: Bridging the gap between annual malaria resurgences in a Sahelian environment.. Am. J. Trop. Med. Hyg..

[11] Monteiro GM, Sonounameto RC, Djogbenou LS, Sedda L: (2025). Spatial and spatio-temporal analysis for malaria hotspot identification: a scoping review protocol.. BMJ Open.

[12] Berthé I, Cissoko M, Kone M, Mbaga D (2025,). Geo-temporal study of clinical malaria in an endemic zone in southern Mali: The case of the Kolondieba health district from 2019 to 2021.. MalariaWorld J..

[13] Cissoko M, Magassa M, Cissé IA, Coulibaly S (2025). Geo-epidemiological risk stratification to select malaria interventions, case of Mali.. PLOS Glob. Public Health.

[14] Institut national de la statistique du Mali (INSAT): Rapport final recensement géographique de la population et de l'habitat, 2009 (RGPH09).. https://tinyurl.com/yucshufw.

[15] KoboToolbox: Data collection using KoboCollect. https://tinyurl.com/bdcwedb9.

[16] Killick R, Fearnhead P, Eckley IA (2012). Optimal detection of changepoints with a linear computational cost.. J. Am. Stat. Assoc..

[17] Zhang SX, Yang GB, Yang J, Wei FN (2024). Global, regional, and national burden of malaria, 1990–2021: Findings from the global burden of disease study 2021.. Decod. Infect. Transm..

[18] Bousema T, Griffin JT, Sauerwein RW, Smith DL, Churcher TS (2012). Hitting hotspots: Spatial targeting of malaria for control and elimination.. PLOS Med..

[19] Union Africain: La technologie gene drive pour la lutte contre le paludisme et son elimination en Afrique; (2021). https://tinyurl.com/3menyj84.

[20] Lubinda J, Bi Y, Haque U, Lubinda M (2022). Spatio-temporal monitoring of health facility-level malaria trends in Zambia and adaptive scaling for operational intervention.. Commun. Med. (Lond)..

[21] Cissoko M, Magassa M, Sanogo V, Koné A (2023). P0-35 Stratification du paludisme au Mali à l’échelle aire de santé et ciblage des interventions.. Santé Publique.

[22] Stresman G, Bousema T, Cook J: (2019). Malaria hotspots: Is there epidemiological evidence for fine-scale spatial targeting of interventions?. Trends Parasitol..

[23] Yamba EI, Fink AH, Badu K, Asare EO (2023). Climate drivers of malaria transmission seasonality and their relative importance in sub‐Saharan Africa.. GeoHealth.

[24] Yao T, Yang X, Ye H, Wang Y (2025). Spatiotemporal patterns and climate-induced macroeconomic burden of malaria in sub-Saharan Africa.. BMC Public Health.

[25] Atusingwize E, Deane K, Musoke D (2025). Social determinants of malaria in low- and middle-income countries: a mixed-methods systematic review.. Malar. J..

[26] Khazaee-Pool M, Moosazadeh M, Asadi-Aliabadi M, Yazdani F (2025). Gender characteristics, social determinants, and seasonal patterns of malaria incidence, relapse, and mortality in Sistan and Baluchistan province and other province of Iran: A systematic review and meta-analysis.. BMC Infect. Dis..

[27] Odhiambo JN, Kalinda C, Macharia PM, Snow RW (2020). Spatial and spatio-temporal methods for mapping malaria risk: a systematic review.. BMJ Glob. Health.

[28] Tam G, Cowling BJ, Maude RJ (2021). Analysing human population movement data for malaria control and elimination.. Malar. J..

